# Release behavior of dissolved carbon species from coal gangue during alkaline hydrothermal treatment

**DOI:** 10.1039/d6ra02471h

**Published:** 2026-08-13

**Authors:** Hui Wang, Ruifang Jia, Yantao Ma, Wen Xue, Fei Du, Jirong Lan, Tianyu Zeng, Min Zhou, Shaoyang Tao, Haobo Hou

**Affiliations:** a Central Southern China Electric Power Design Institute Co., Ltd, China Power Engineering Consulting Group Wuhan 430071 China; b School of Resource and Environmental Science, Wuhan University Wuhan 430072 Hubei China shaoyang.tao@whu.edu.cn hhb-bhh@163.com

## Abstract

Coal gangue is produced in large quantities and may release dissolved carbon during long-term weathering and leaching, yet its carbon mobilization behavior under alkaline hydrothermal conditions remains insufficiently understood. This study investigated the effects of extractant type, particle size, KOH concentration, liquid-to-solid ratio, hydrothermal temperature, and reaction time on the release of dissolved carbon (DC), dissolved organic carbon (DOC), and dissolved inorganic carbon (DIC) from coal gangue. Among the tested extractants, KOH showed the highest extraction capacity and was selected as the alkaline medium for subsequent experiments. Orthogonal optimization identified 180 °C, 10 mL g^−1^ liquid-to-solid ratio, 0.75 mol per L KOH, and 18 h as the optimal conditions, under which DC, DOC, and DIC releases reached 24.58, 18.43, and 6.15 mg g^−1^, respectively. Range analysis and ANOVA indicated that hydrothermal temperature and liquid-to-solid ratio were the dominant factors within the investigated range, while KOH provided the alkaline environment required for carbon mobilization. EEM analysis showed that the released DOC mainly exhibited humic-like and fulvic-like fluorescence characteristics. Structural characterization further revealed aluminosilicate framework transformation, pore-structure enlargement, surface chemical changes, and the formation of K-bearing silicate/aluminosilicate crystalline phases after alkaline hydrothermal treatment. These results indicate that dissolved carbon release from coal gangue is controlled by the coupled effects of alkaline attack, mineral phase transformation, pore-structure opening, and organic carbon liberation. This study provides new insight into dissolved carbon mobilization from coal gangue and offers a basis for regulating carbon release during the hydrothermal utilization of coal-based solid wastes.

## Abstract 2

Optimized alkaline hydrothermal treatment drives the differentiated release of DC, DOC, and DIC from coal gangue through pore opening, aluminosilicate transformation, and K-bearing phase formation.

## Graphical abstract

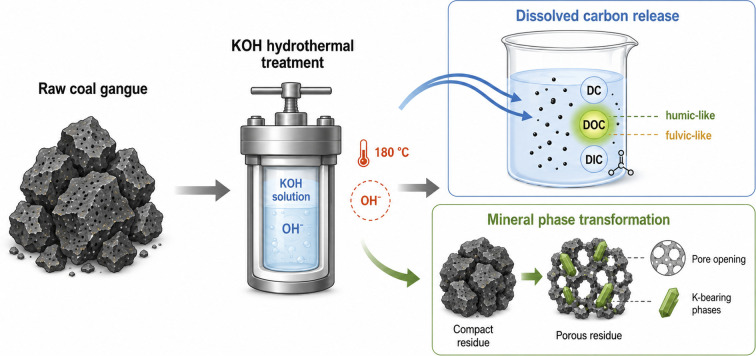

## 1. Introduction

Coal gangue is one of the major solid wastes generated during coal mining and washing processes.^1–4^ Its long-term accumulation occupies large areas of land and may pose environmental risks through the release of harmful substances into surrounding water, soil, and air during weathering and leaching.^5–7^ As a coal-rich country, China has accumulated a large amount of coal gangue, making its resource utilization and harmless disposal an important issue in solid-waste management. In addition to mineral components, coal gangue contains carbonaceous substances, including organically bound carbon and inorganic carbonates.^7–10^ Dissolved organic carbon (DOC), as an active fraction of organic matter, plays an important role in nutrient cycling, soil structure improvement, and fertility enhancement.^11–13^ Therefore, understanding and regulating the release of dissolved carbon species from coal gangue may provide a potential pathway for its value-added utilization, especially in soil improvement and ecological restoration.

Previous studies have explored carbon release or organic matter transformation from coal gangue using conventional extraction, biological degradation, or oxidative cracking methods.^14–16^ Hydrothermal treatment has also been applied to promote mineral activation and resource conversion of coal gangue and other solid wastes.^17–19^ In parallel, alkaline activation can enhance aluminosilicate dissolution, mineral reconstruction, and the synthesis of silicate- or zeolite-like functional materials from coal gangue.^20–23^ In addition, carbon separation and decarbonization of coal-based solid wastes have recently received increasing attention.^24^ However, most existing studies have focused on mineral transformation, fertilizer preparation, adsorbent synthesis, pollutant removal, or carbon separation, while the differentiated release behavior of dissolved carbon fractions, including dissolved carbon (DC), DOC, and dissolved inorganic carbon (DIC), remains insufficiently understood.

Based on these research gaps, this study used coal gangue from Ordos, Inner Mongolia, as the raw material and selected KOH as an effective alkaline hydrothermal medium through extractant screening. The effects of extractant type, particle size, KOH concentration, liquid-to-solid ratio, hydrothermal temperature, and reaction time on the release of DC, DOC, and DIC were systematically investigated. Orthogonal experiments, range analysis, and ANOVA were used to optimize the process and evaluate the significance of operational factors. Furthermore, EEM fluorescence spectroscopy and multi-scale characterization techniques, including SEM-EDS, XRD, FT-IR, N_2_ adsorption–desorption, and XPS, were employed to reveal the DOC characteristics and mineral-structure changes during alkaline hydrothermal treatment. This study aims to clarify the coupling relationship between dissolved carbon release and mineral transformation, providing a theoretical basis for regulating carbon mobilization during the hydrothermal utilization of coal gangue.

## 2. Materials and methods

### 2.1 Sample collection and preparation

Coal gangue used in this study was collected from a coal mine in Ordos, Inner Mongolia, China. The as-received gangue appeared as gray–black blocks and was first crushed, ground, and sieved to obtain fractions of defined particle sizes. The pH of the gangue leachate was measured according to standard leaching procedures, while the loss on ignition (LOI) was determined following GB/T 35986-2018 by heating the sieved sample at 800 °C in a muffle furnace.

The major elemental composition of the coal gangue was analyzed using X-ray fluorescence (XRF). As shown in Table 1, SiO_2_ and Al_2_O_3_ were the predominant components, together accounting for 62.3% of the measured composition, while the LOI reached 33.0%, reflecting the relatively high volatile and carbonaceous content of this material. The gangue leachate exhibited a slightly acidic pH of 6.77.

**Table 1:** Main chemical composition and loss on ignition of coal gangue (%)

SiO_2_	Al_2_O_3_	Fe_2_O_3_	CaO	MgO	K_2_O	Na_2_O	TiO_2_	SO_3_	LOI
32.4	29.9	0.6	0.8	0.2	0.1	0.8	0.7	1.6	33.0

### 2.2 Hydrothermal leaching experiments

Hydrothermal leaching experiments were performed to investigate the release behavior of dissolved carbon species under alkaline conditions. Initially, seven different leaching agents—deionized water, CaO, KCl, H_3_PO_4_, HNO_3_, NaOH, and KOH—were screened to evaluate their effectiveness in extracting dissolved carbon from coal gangue.

After identifying KOH as the most effective leaching agent, the effects of particle size (0.074–0.15, 0.15–0.25, 0.25–1, 1–2, and 2–5 mm), KOH concentration (0, 0.25, 0.5, 0.75, and 1 mol L^−1^), liquid-to-solid ratio (2, 4, 6, 8, and 10 mL g^−1^), hydrothermal temperature (100, 120, 140, 160, and 180 °C), and reaction time (1, 6, 12, 18, and 24 h) were systematically investigated.

For each experiment, a measured amount of coal gangue was loaded into a Teflon-lined stainless-steel autoclave, followed by the addition of a predetermined volume of KOH solution. The mixture was stirred homogeneously and then sealed for hydrothermal treatment in an oven under the designated temperature and time. After cooling to room temperature, the resulting suspension was centrifuged, filtered, and diluted for subsequent analyses.

The concentrations of dissolved carbon (DC), dissolved organic carbon (DOC), and dissolved inorganic carbon (DIC) in the leachates were quantified using a TOC analyzer (TOC-2000, Yuanxi Instruments, China).

### 2.3 Orthogonal experimental design

To optimize the hydrothermal leaching process, a four-factor, three-level orthogonal design was employed. The factors included hydrothermal temperature (*A*), liquid-to-solid ratio (*B*), KOH concentration (*C*), and reaction time (*D*), with the corresponding levels listed in Table 2. Experimental results, range analysis, and analysis of variance (ANOVA) were used to determine the relative influence of each factor on DC, DOC, and DIC release and to identify the optimal combination of parameters. Validation experiments were conducted under the optimal condition to verify the reliability of the optimized parameter combination.

**Table 2:** Experimental factors and levels

Factors	*A*: temperature (°C)	*B*: liquid–solid ratio (mL g^−1^)	*C*: KOH concentration (mol L^−1^)	*D*: reaction time (h)
Level 1	140	6	0.50	12
Level 2	160	8	0.75	18
Level 3	180	10	1.00	24

### 2.4 Statistical analysis

All replicated experiments were expressed as mean ± standard deviation. Error bars in the figures represent standard deviations of triplicate experiments. ANOVA was performed using a main-effects model to evaluate the statistical significance of the factors in the orthogonal experiments, and differences were considered significant at *p* < 0.05. Since the L9(3^4^) orthogonal design was mainly used to evaluate main effects, center points and independent interaction terms were not included.

### 2.5 Characterization techniques

A suite of analytical methods was employed to characterize the elemental composition, mineral phases, functional groups, and microstructural changes of coal gangue before and after hydrothermal treatment. The elemental composition of the raw sample was determined using X-ray fluorescence spectroscopy (XRF, S4 PIONEER, Bruker AXS). Functional groups were analyzed by Fourier-transform infrared spectroscopy (FT-IR, Nicolet 5700, Thermo Fisher Scientific) using the KBr pellet method, with spectra collected in the range of 400–4000 cm^−1^. Mineralogical transformations were examined using X-ray diffraction (XRD, D8 Advance, Bruker) with Cu Kα radiation over a scanning range of 10–80° 2*θ*, and phase identification was performed using Jade software. The morphology and elemental distribution of the samples were observed by scanning electron microscopy coupled with energy-dispersive spectroscopy (SEM-EDS, QUANTA, FEI). The pore structure was analyzed by N_2_ adsorption–desorption measurements, and the BET surface area, pore volume, and pore-size distribution were obtained from the adsorption data according to standard physisorption analysis principles.^25^ Surface elemental composition and chemical states were analyzed by X-ray photoelectron spectroscopy (XPS). EEM fluorescence spectroscopy was used to characterize the optical properties of DOC in the leachates, following the fluorescence-based identification of dissolved organic matter.^26^

The crystallinity index was calculated from the XRD patterns using the area method. After background correction and peak fitting, the crystallinity index was determined as the ratio of crystalline peak area to total diffraction area. The average crystallite size was estimated using the Debye–Scherrer equation:^27^
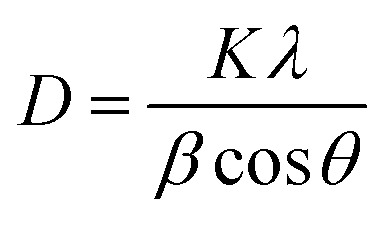
where *D* is the crystallite size, *K* is the shape factor of 0.90, *λ* is the wavelength of Cu Kα radiation (0.15406 nm), *β* is the full width at half maximum (FWHM) in radians, and *θ* is the Bragg angle. The average crystallite size was calculated from selected characteristic diffraction peaks.

## 3. Results and discussion

### 3.1 Effects of operational parameters on dissolved carbon release

**Fig. 1:**
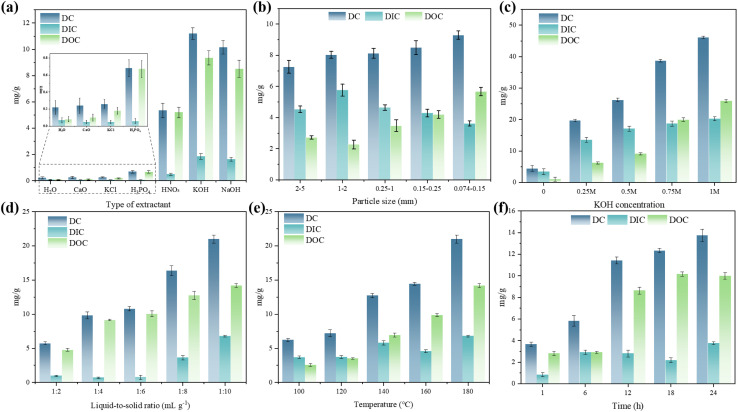
Effects of operational parameters on the release of dissolved carbon species from coal gangue. (a) Type of extractant; (b) particle size; (c) KOH concentration; (d) liquid-to-solid ratio; (e) hydrothermal temperature; and (f) reaction time. DC, dissolved carbon; DOC, dissolved organic carbon; DIC, dissolved inorganic carbon. Error bars represent standard deviations of triplicate experiments.

The effects of operational parameters on the release of dissolved carbon species from coal gangue are shown in Fig. 1. Among the tested extractants, KOH showed the highest extraction capacity (Fig. 1a), with DC and DOC release reaching approximately 10.0 and 8.1 mg g^−1^, respectively. NaOH also promoted carbon release effectively, whereas H_2_O, CaO, KCl, and H_3_PO_4_ showed limited extraction effects, and HNO_3_ promoted carbon release to a lesser extent than the strong alkaline extractants. These results suggest that strong alkaline conditions were more favorable for dissolved carbon release from coal gangue, which is consistent with previous studies showing that alkaline activation can enhance aluminosilicate dissolution and mineral transformation in coal gangue.^19,22,23^ Therefore, KOH was selected as an effective alkaline medium for the subsequent hydrothermal experiments.

Particle size had a clear influence on carbon release (Fig. 1b). The release of DC and DOC generally increased with decreasing particle size, and the finest fraction of 0.074–0.15 mm showed the highest release. This can be attributed to the increased exposed surface area and shortened diffusion path of fine particles, which enhanced the contact between coal gangue and the alkaline solution. Similar effects of particle-size reduction or mechanical activation on mineral reactivity have also been reported for coal gangue and aluminosilicate materials.^20,21^

KOH concentration and liquid-to-solid ratio further affected the release behavior (Fig. 1c and d). Increasing KOH concentration from 0 to 1.0 mol L^−1^ markedly increased DC, DOC, and DIC release, indicating that OH^−^ promoted the decomposition of mineral-associated structures and the mobilization of dissolved carbon species. This is in agreement with previous reports that alkaline activation can facilitate aluminosilicate dissolution, framework reconstruction, and the formation of silicate- or zeolite-like products from coal gangue.^19,22,23^ A higher liquid-to-solid ratio also enhanced carbon release, probably due to improved alkaline solution supply and mass transfer.

Hydrothermal temperature and reaction time also played important roles (Fig. 1e and f). Increasing temperature from 100 to 180 °C continuously promoted DC and DOC release, indicating enhanced hydrothermal reaction and carbon mobilization at elevated temperature. Hydrothermal treatment has been reported to accelerate dissolution, hydrolysis, and structural rearrangement reactions during solid-waste conversion.^17,18^ With increasing reaction time, DC and DOC release increased rapidly at the early stage and then tended to level off. DOC release increased markedly before 18 h, whereas extending the reaction time to 24 h resulted in no obvious additional improvement. Therefore, KOH concentration, liquid-to-solid ratio, hydrothermal temperature, and reaction time were identified as the key factors controlling dissolved carbon release from coal gangue.

### 3.2 Orthogonal experiment optimization

To optimize the alkaline hydrothermal conditions for dissolved carbon release, a four-factor, three-level orthogonal experiment was conducted. Orthogonal experimental design is an efficient method for evaluating the main effects of multiple factors with a reduced number of experimental runs.^28^ The investigated factors were hydrothermal temperature (*A*), liquid-to-solid ratio (*B*), KOH concentration (*C*), and reaction time (*D*), and the experimental results are summarized in Table 3.

**Table 3:** Orthogonal experimental results^a^

Test no.	*A*	*B*	*C*	*D*	DC (mg g^−1^)	DOC (mg g^−1^)	DIC (mg g^−1^)
1	1	1	1	1	5.97 ± 0.11	4.53 ± 0.08	1.44 ± 0.03
2	1	2	2	2	8.98 ± 0.16	7.14 ± 0.12	1.84 ± 0.04
3	1	3	3	3	11.21 ± 0.20	8.23 ± 0.14	2.98 ± 0.06
4	2	1	2	3	13.86 ± 0.21	11.07 ± 0.16	2.79 ± 0.05
5	2	2	3	1	9.18 ± 0.15	7.34 ± 0.11	1.85 ± 0.04
6	2	3	1	2	20.52 ± 0.29	15.55 ± 0.22	4.97 ± 0.07
7	3	1	3	2	17.42 ± 0.25	14.03 ± 0.19	3.39 ± 0.06
8	3	2	1	3	15.80 ± 0.22	12.30 ± 0.17	3.50 ± 0.05
9	3	3	2	1	23.41 ± 0.32	18.06 ± 0.24	5.35 ± 0.08

a *A*, *B*, *C*, and *D* represent hydrothermal temperature, liquid-to-solid ratio, KOH concentration, and reaction time, respectively. Values are expressed as mean ± standard deviation (*n* = 3). DC, DOC, and DIC represent dissolved carbon, dissolved organic carbon, and dissolved inorganic carbon, respectively.

Range analysis was performed to evaluate the relative influence of each factor (Table 4). For DC and DOC release, factor *A* showed the largest range value, followed by factor *B*, indicating that hydrothermal temperature and liquid-to-solid ratio were the dominant factors. For DIC release, factor *B* showed a slightly stronger influence than factor *A*. The optimal combination for DC, DOC, and DIC release was consistently determined as *A*_3_*B*_3_*C*_2_*D*_2_, corresponding to 180 °C, 10 mL g^−1^ liquid-to-solid ratio, 0.75 mol per L KOH, and 18 h.

**Table 4:** Range analysis^a^

Index	Metric	*A*	*B*	*C*	*D*	Optimal combination
DC	*K*_1_	8.72	12.42	14.10	12.85	*A*_3_ *B*_3_ *C*_2_ *D*_2_
	*K*_2_	14.52	11.32	15.42	15.64	
	*K*_3_	18.88	18.38	12.61	13.62	
	Range (*R*)	10.16	7.06	2.81	2.79	
DOC	*K*_1_	6.63	9.88	10.79	9.98	*A*_3_ *B*_3_ *C*_2_ *D*_2_
	*K*_2_	11.32	8.92	12.09	12.24	
	*K*_3_	14.80	13.95	9.87	10.53	
	Range (*R*)	8.16	5.02	2.22	2.26	
DIC	*K*_1_	2.09	2.54	3.30	2.88	*A*_3_ *B*_3_ *C*_2_ *D*_2_
	*K*_2_	3.20	2.40	3.33	3.40	
	*K*_3_	4.08	4.43	2.74	3.09	
	Range (*R*)	1.99	2.04	0.59	0.21	

a *K*_1_, *K*_2_, and *K*_3_ represent the average response values of each factor at levels 1, 2, and 3, respectively. *R* represents the range value, which was used to evaluate the relative influence of each factor on carbon release.

ANOVA was further conducted using a main-effects model to evaluate the statistical significance of each factor (Table 5). Hydrothermal temperature, liquid-to-solid ratio, KOH concentration, and reaction time all significantly affected DC, DOC, and DIC release (*p* < 0.001). Hydrothermal temperature contributed most to DC and DOC release, with contributions of 58.35% and 63.19%, respectively, followed by liquid-to-solid ratio. For DIC release, liquid-to-solid ratio showed the highest contribution of 52.27%, followed by hydrothermal temperature. These results were consistent with the range analysis, indicating that hydrothermal temperature and liquid-to-solid ratio were the dominant operational factors, while KOH concentration acted as an essential chemical driving factor for alkaline dissolution and carbon mobilization.

**Table 5:** ANOVA results for dissolved carbon release in the orthogonal experiment^a^

Response	Factor	df	*F* value	*p* value	Contribution/%
DC	*A*	2	4761.17	<0.001	58.35
DC	*B*	2	2647.05	<0.001	32.44
DC	*C*	2	363.32	<0.001	4.45
DC	*D*	2	379.77	<0.001	4.65
DOC	*A*	2	5457.02	<0.001	63.19
DOC	*B*	2	2312.39	<0.001	26.78
DOC	*C*	2	405.56	<0.001	4.70
DOC	*D*	2	452.27	<0.001	5.24
DIC	*A*	2	2929.34	<0.001	40.37
DIC	*B*	2	3792.36	<0.001	52.27
DIC	*C*	2	323.84	<0.001	4.46
DIC	*D*	2	200.84	<0.001	2.77

a *A*, *B*, *C*, and *D* represent hydrothermal temperature, liquid-to-solid ratio, KOH concentration, and reaction time, respectively. ANOVA was performed using a main-effects model without interaction terms. Contribution was calculated as the ratio of each factor's sum of squares to the corrected total sum of squares.

Verification experiments under the optimal condition yielded DC, DOC, and DIC releases of 24.58, 18.43, and 6.15 mg g^−1^, respectively. These values were higher than those obtained in the single-factor screening experiments, confirming the effectiveness of the optimized parameter combination. The L9(3^4^) orthogonal design was mainly used to evaluate main effects; therefore, center points and independent interaction terms were not included.

### 3.3 Optical characteristics and source identification of released DOC

**Fig. 2:**
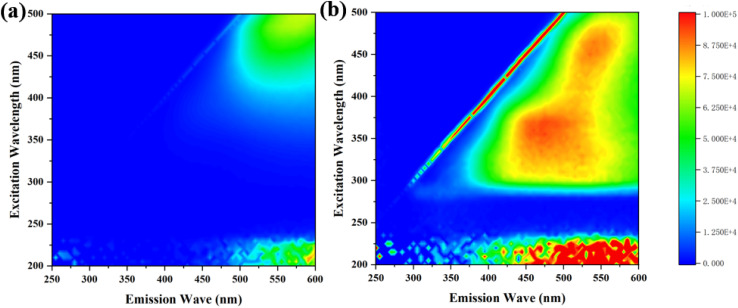
EEM fluorescence spectra of dissolved organic matter released from coal gangue. (a) Water extract of raw coal gangue; (b) alkaline hydrothermal leachate obtained under the optimal KOH treatment condition.

To further identify the properties and possible source of the released DOC, EEM fluorescence spectroscopy was performed for the water extract of raw coal gangue and the alkaline hydrothermal leachate. As shown in Fig. 2, the water extract showed only weak fluorescence signals, whereas the alkaline hydrothermal leachate exhibited much stronger and broader fluorescence regions. According to previous EEM-based DOM studies, fluorescence signals in these regions are generally associated with humic-like and fulvic-like dissolved organic matter.^26^ This result indicates that alkaline hydrothermal treatment promoted the release of humic-like and fulvic-like DOC from coal gangue.

The weak fluorescence of the water extract suggests that only a small amount of water-extractable organic matter was present in raw coal gangue. In contrast, the enhanced fluorescence intensity after alkaline hydrothermal treatment indicates that KOH promoted the liberation of organic carbon associated with the mineral matrix. Previous studies have shown that KOH-assisted hydrothermal treatment can extract humic acid from lignite, while alkali-thermal humification can transform dissolved organic matter into more humified components.^29,30^ Therefore, the broadened fluorescence regions observed in the alkaline hydrothermal leachate may be attributed to both the release of native or mineral-associated organic matter and partial transformation into more soluble humic-like and fulvic-like components.

### 3.4 Mineralogical, textural, and surface-chemical evolution of coal gangue

#### 3.4.1 Morphological, mineralogical, and pore-structure evolution

**Fig. 3:**
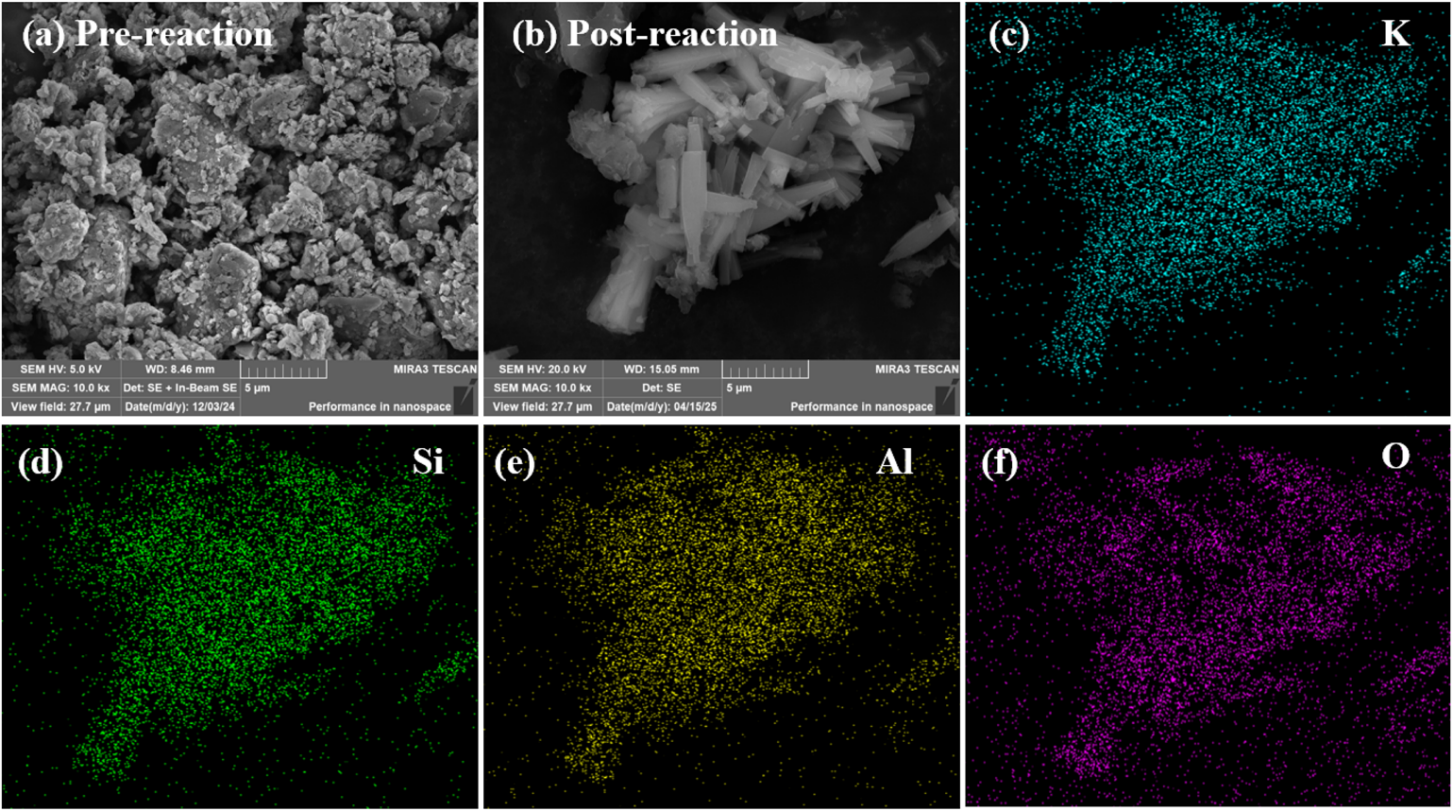
Morphology and elemental distribution of coal gangue before and after alkaline hydrothermal treatment. (a) SEM image of raw coal gangue; (b) SEM image of KOH-treated residue; EDS elemental mapping of (c) K, (d) Si, (e) Al, and (f) O in the KOH-treated residue.

SEM-EDS analysis was conducted to examine the morphological evolution and elemental distribution of coal gangue before and after alkaline hydrothermal treatment. As shown in Fig. 3a, the raw coal gangue consisted of compact and aggregated particles. After KOH hydrothermal treatment, rod-like or prismatic crystals appeared on the residue surface (Fig. 3b), indicating that dissolution–reprecipitation and secondary crystallization occurred during the reaction. Similar morphology changes have been reported during the hydrothermal or alkali-hydrothermal conversion of coal gangue into zeolite-like aluminosilicate materials.^31,37,38^ The EDS mapping images showed that K, Si, Al, and O were spatially co-distributed in the treated residue (Fig. 3c–f), suggesting that K was incorporated into the aluminosilicate/silicate-rich region rather than only remaining as isolated surface residue.

**Fig. 4:**
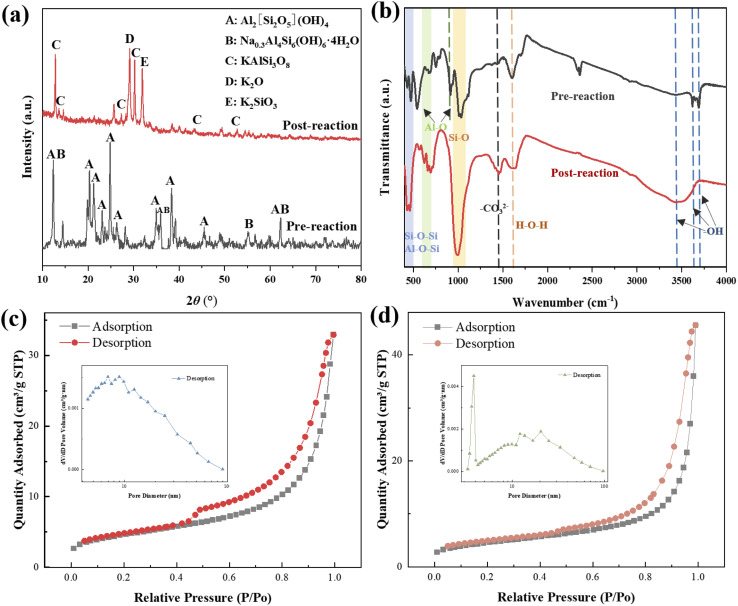
Mineralogical, functional-group, and pore-structure evolution of coal gangue before and after alkaline hydrothermal treatment. (a) XRD patterns; (b) FT-IR spectra; N_2_ adsorption–desorption isotherms and BJH pore-size distributions of (c) raw coal gangue and (d) KOH-treated residue.

The XRD patterns further confirmed the mineralogical transformation (Fig. 4a). After alkaline hydrothermal treatment, the characteristic peaks of the original clay minerals were weakened, while new diffraction peaks assigned to K-bearing aluminosilicate and silicate phases, such as KAlSi_3_O_8_ and K_2_SiO_3_, appeared. This indicates that the original aluminosilicate framework was partially depolymerized and subsequently reconstructed into secondary crystalline phases under alkaline hydrothermal conditions.^19,31,35,38^ As shown in Table 6, the crystallinity index increased from 31.9% to 44.3%, and the average crystallite size slightly increased from 32.94 ± 4.72 nm to 36.60 ± 5.71 nm after treatment. These results suggest that alkaline hydrothermal treatment promoted mineral phase transformation and the formation of K-bearing secondary crystalline phases, rather than simply causing amorphization of the solid residue.

**Table 6:** Crystallinity index and average crystallite size of coal gangue before and after alkaline hydrothermal treatment^a^

Sample	Crystallinity index/%	Average crystallite size/nm
Pre-reaction	31.9	32.94 ± 4.72
Post-reaction	44.3	36.60 ± 5.71

a The crystallinity index was calculated using the area method. The average crystallite size was estimated using the Debye–Scherrer equation based on selected characteristic diffraction peaks.

FT-IR spectra also revealed changes in the aluminosilicate framework (Fig. 4b). Compared with raw coal gangue, the KOH-treated residue showed variations in Si–O, Al–O, Si–O–Si, and Al–O–Si vibration bands, accompanied by weakened hydroxyl-related bands. These bands are commonly used to identify structural changes in clay minerals and aluminosilicate materials.^32,33,36^ The observed spectral changes suggest the destruction of clay-mineral structures and the reorganization of silicate/aluminosilicate units during alkaline hydrothermal treatment.

The pore-structure evolution was evaluated by N_2_ adsorption–desorption analysis (Fig. 4c and d). Both samples exhibited type IV-like isotherms with hysteresis loops, indicating mesoporous characteristics according to the IUPAC classification.^25^ As summarized in Table 7, the BET surface area changed only slightly from 16.74 to 16.54 m^2^ g^−1^ after alkaline hydrothermal treatment. In contrast, the total pore volume increased from 0.04796 to 0.06832 cm^3^ g^−1^, and the BJH average pore diameter increased from 14.58 to 20.98 nm. These results indicate that alkaline hydrothermal treatment mainly enlarged existing mesopores and interparticle pores, rather than generating abundant new micropores. The enlarged pore structure may facilitate alkaline solution penetration into the mineral matrix and enhance the accessibility of mineral-associated organic carbon.

**Table 7:** Textural properties of coal gangue before and after alkaline hydrothermal treatment^a^

Sample	BET surface area/m^2^ g^−1^	Total pore volume/cm^3^ g^−1^	BJH average pore diameter/nm
Raw coal gangue	16.74	0.04796	14.58
KOH-treated residue	16.54	0.06832	20.98

a Total pore volume and BJH average pore diameter were calculated from the adsorption branch.

#### 3.4.2 Surface chemical characteristics revealed by XPS

**Fig. 5:**
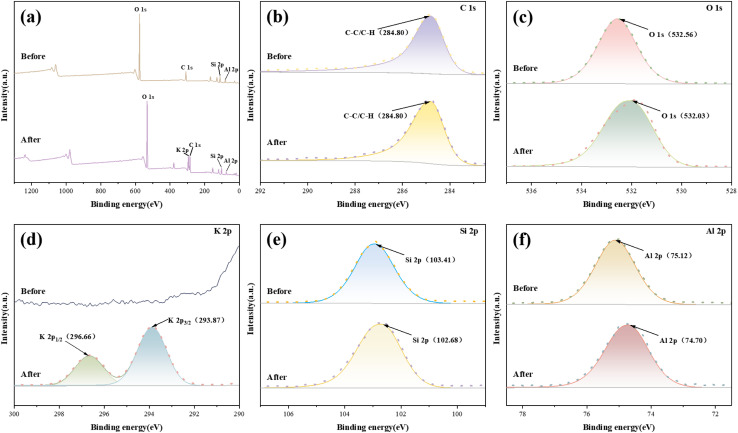
XPS spectra of coal gangue before and after alkaline hydrothermal treatment. (a) Survey spectra; high-resolution spectra of (b) C 1s, (c) O 1s, (d) K 2p, (e) Si 2p, and (f) Al 2p.

XPS analysis was used to further examine the surface chemical characteristics of raw coal gangue and the KOH-treated residue. As shown in Table 8 and Fig. 5, the surface K content increased from 0.39 to 7.35 at% after alkaline hydrothermal treatment, while the Si and Al contents decreased from 13.44 and 12.67 at% to 11.70 and 9.67 at%, respectively. This result indicates that K-containing species were present on the treated residue surface, accompanied by changes in the surface aluminosilicate composition.

**Table 8:** Surface elemental composition of raw coal gangue and KOH-treated residue determined by XPS

Element	Raw coal gangue/at%	KOH-treated residue/at%
C 1s	20.28	25.47
O 1s	53.23	45.81
Si 2p	13.44	11.70
Al 2p	12.67	9.67
K 2p	0.39	7.35

The high-resolution spectra showed that the O 1s, Si 2p, and Al 2p peaks shifted from 532.56, 103.41, and 75.12 eV to 532.03, 102.68, and 74.70 eV, respectively. Since the binding energies of Si 2p, Al 2p, and O 1s are sensitive to the local coordination environment of aluminosilicate materials, these shifts suggest that the local bonding environments of Si, Al, and O were altered after alkaline hydrothermal treatment.^34^ The appearance and enrichment of K 2p further support the incorporation of K-containing species into the transformed silicate/aluminosilicate surface. The C 1s spectra were mainly dominated by C–C/C–H at 284.80 eV. Overall, the XPS results provide supporting evidence for changes in the surface chemical environment of coal gangue after KOH hydrothermal treatment.

### 3.5 Proposed mechanism of dissolved carbon release during alkaline hydrothermal treatment

Based on the above results, the release of dissolved carbon species from coal gangue during alkaline hydrothermal treatment can be interpreted as a mineral-coupled process. Under strong alkaline conditions, OH^−^ attacks Si–O–Al and Si–O–Si linkages in the aluminosilicate framework, leading to the partial depolymerization of clay-mineral structures. This process weakens the mineral matrix and promotes the exposure of mineral-associated carbon species. Meanwhile, the increase in pore volume and average pore diameter improves the accessibility of alkaline solution to the internal mineral surface, further facilitating carbon mobilization.

The EEM results indicated that the released DOC mainly exhibited humic-like and fulvic-like fluorescence characteristics, suggesting the liberation of native or mineral-associated organic matter from coal gangue. XRD, FT-IR, SEM-EDS, BET, and XPS analyses further showed that alkaline hydrothermal treatment induced mineral phase transformation, pore-structure enlargement, and changes in the surface chemical environment. The formation of K-bearing silicate/aluminosilicate crystalline phases indicates that the dissolved silicate and aluminate species may partially recombine with K-containing species during the reaction. Therefore, dissolved carbon release is not controlled by a single dissolution process, but by the coupled effects of aluminosilicate framework depolymerization, pore-structure opening, organic carbon liberation, and secondary mineral formation.

## 4. Conclusion

This study investigated the release behavior of dissolved carbon species from coal gangue during alkaline hydrothermal treatment. Among the tested extractants, KOH showed the highest extraction capacity and was selected as the alkaline medium for subsequent experiments. The optimal condition was determined as 180 °C, 10 mL g^−1^ liquid-to-solid ratio, 0.75 mol per L KOH, and 18 h, under which the releases of DC, DOC, and DIC reached 24.58, 18.43, and 6.15 mg g^−1^, respectively. Range analysis and ANOVA indicated that hydrothermal temperature and liquid-to-solid ratio were the dominant factors within the investigated range, while KOH provided the alkaline environment required for carbon mobilization.

EEM analysis showed that the released DOC mainly exhibited humic-like and fulvic-like fluorescence characteristics, suggesting the mobilization of native or mineral-associated organic matter. Structural characterization further demonstrated that alkaline hydrothermal treatment induced aluminosilicate framework depolymerization, mineral phase transformation, pore-structure enlargement, and changes in the surface chemical environment. The formation of K-bearing silicate/aluminosilicate crystalline phases indicated that secondary mineral reconstruction also occurred during the reaction.

Overall, dissolved carbon release from coal gangue was controlled by the coupled effects of alkaline attack, mineral framework transformation, pore-structure opening, and organic carbon liberation. These findings improve the understanding of carbon mobilization from coal gangue and provide a basis for regulating dissolved carbon release during the hydrothermal utilization of coal-based solid wastes.

## Author contributions

Hui Wang: investigation, data curation, writing – original draft. Ruifang Jia: methodology, formal analysis. Yantao Ma: validation, visualization. Wen Xue: resources, project administration. Fei Du: investigation, data curation. Jirong Lan: formal analysis, visualization. Tianyu Zeng: methodology, validation. Min Zhou: supervision, resources. Shaoyang Tao: conceptualization, writing – review & editing, supervision. Haobo Hou: conceptualization, funding acquisition, supervision.

## Conflicts of interest

The authors declare that they have no known competing financial interests or personal relationships that could have appeared to influence the work reported in this paper.

## Data Availability

All data supporting the findings of this study are included within the article.
